# Oral Status in Pregnant Women from Post-Industrial Areas of Upper Silesia in Reference to Occurrence of: Preterm Labors, Low Birth Weight and Type of Labor

**DOI:** 10.3390/healthcare8040528

**Published:** 2020-12-01

**Authors:** Marta Katarzyńska-Konwa, Izabela Obersztyn, Agata Trzcionka, Katarzyna Mocny-Pachońska, Bartosz Mosler, Marta Tanasiewicz

**Affiliations:** Department of Conservative Dentistry with Endodontics, Faculty of Medical Sciences in Zabrze, Medical University of Silesia, Plac Akademicki 17, 41-902 Bytom, Poland; mkatarzynska88@gmail.com (M.K.-K.); endobytom@poczta.onet.pl (I.O.); kpachonska@sum.edu.pl (K.M.-P.); bartosz.mosler@sum.edu.pl (B.M.); martatanasiewicz@sum.edu.pl (M.T.)

**Keywords:** oral hygiene, pregnancy, periodontal status

## Abstract

Increased levels of steroid hormones, action of local irritants and the lack of proper hygiene measures are of great importance in the development of dental caries, gingivitis and inflammation of the periodontal area in pregnant women. The aim of the study was to evaluate the state of oral hygiene and the periodontal area is such a population and assess the effectiveness of performed hygiene treatments and analyse changes in hygiene habits after oral hygiene instructions. The study was performed in two parts on a group of 50 pregnant women. The first part took place between the 14th and 17th week of pregnancy. The control study was conducted between the 27th and 30th week of pregnancy. Patients were subjected to a dental examination. Poor oral hygiene was observed among the examined patients. After the first examination, oral hygiene instruction was provided to 25 randomly selected pregnant patients. The effect of periodontal diseases on the time of labor was observed. Oral hygiene instructions significantly affected the state of the periodontal area of pregnant women for whom it was performed. It was confirmed that the advancement of pregnancy influences deterioration of the periodontium and also term of childbirth. Undoubtedly, pregnant women receive insufficient dental care. Priority should be given to dental care education of for pregnant women and alleviating the impact of oral diseases on the organism of a pregnant woman.

## 1. Introduction

Pregnancy is a special time in a woman’s life, during which a number of physiological changes take place in order for the woman to accommodate the developing and growing fetus. The oral cavity may reflect a variety of disorders and the condition of the oral cavity doesn’t remain indifferent to the changes taking place in the pregnant body, and their causes are not clearly explained. Contemporary literature emphasizes the multifactorial nature of pathogenesis of developing periodontitis in pregnant women, for which the mechanism of origin is mainly related to the hormonal, genetic and behavioral changes [1,2]. Fluctuations in the level of steroid hormones, their effects on periodontal cells and the composition of biofilm are considered the basic factor modulating changes in the oral cavity. Studies show that increased levels of steroid hormones also effect the composition of subgingival calculus. The specific changes in the biofilm, which becomes richer in anaerobic Gram (–) *Prevotella* bacteria is an important causative factor in gingivitis. Their growth may be associated with the fact that steroid hormones are substitutes for vitamin K, which is an important growth factor for these bacteria. Additionally, slower keratinization of the peripheral gingival epithelium, increased depth of gingival pockets, which is a potential place for their growth, indirectly contributes to the increase in their quantity [3].

Already in 1980s, Kornman and Loesche described the increase in the number of *Prevotella intermedia* in the second trimester of pregnancy [4]. Contemporary studies by Betley-Gromada showed the presence of anaerobic Gram (+) and Gram (–) bacteria in the inflammatory secretion taken from gingival fissures in pregnant women with symptoms of periodontal inflammation. The author stated that bacteria showing very strong and moderate influence on the development of periodontium inflammation dominated [5]. In addition, the changes appearing in the immune system and disruption of the body’s defensive response determine the development of inflammatory conditions within the periodontal tissues [1]. The most frequently observed changes in the oral cavity of pregnant women manifest themselves as reddening of the gingival mucosa. The clinical picture is defined as gingivitis gravidarum, which was defined by the American Academy of Periodontology in 1999 as gingivitis associated with dental plaque, modified by general factors such as steroid hormones. The frequency of gum inflammatory changes during pregnancy ranges from 35% to 100%. The severity of symptoms is observed between 14 and 30 weeks of pregnancy [6]. Very rarely does gingivitis in pregnant women develop into periodontitis, which is confirmed by the loss of the connective tissue attachment. This fact is associated with the suppressive action of progesterone on gingival fibroblasts, which inhibits the production of MMP metalloproteinaeses, the enzyme responsible for destruction of collagen fibers [6].

Pregnancy tumors (epulis gravidarum) are another characteristic disease entity that may appear in the oral cavity of pregnant women. They were described by Hullihen in 1844 as hemangioma hyperplasia, capillary angiomas. Their incidence in pregnant women ranges from 0.5% to 5% [7]. The causes of their formation are associated with the response of periodontal tissues to chronic irritation by plaque, calculus, overhanging fillings, as well as micro-injuries resulting from tooth brushing or flossing. Additional impulses inducing the formation of pregnancy tumors are high concentrations of steroid hormones. The clinical manifestation is a mushroom-shaped, easily bleeding exaggeration. The colouring depends on the vascularization and can range from vivid red to purple [8]. The tongue, palate, cheek mucosa and lips are also potential sites where pregnancy tumors may be located [9]. Pregnancy is a period characterized by high intensity of caries [10]. This may by associated with modification of dietary habits and insufficient hygienic procedures performed in the oral cavity. Caries are formed by the action of acids produced by caries-forming bacteria that metabolise carbohydrates from the diet, which lead to the demineralization of hard dental tissues. Sometimes increased consumption of products that are a source of simple carbohydrates, between meals, promotes an increase in the number of cariogenic bacteria i.e., *Streptococcus mutans*. In addition, early pregnancy symptoms, such as morning nausea and vomiting, gastroesophageal reflux caused by the pressure of the growing uterus on the stomach, make the oral cavity more frequently exposed to acidic nutrients, which may result in the appearance of demineralization changes on tooth surfaces. Saliva plays an important role in pathophysiology of caries and oral inflammation during pregnancy. More acidic pH and lower buffer capacity are observed. The amount of mucins increases, causing saliva to become less watery and more viscous [11]. All these physiological changes promote the build-up of plaque on tooth surfaces and contribute to the maintenance of an acid environment it the oral cavity of pregnant women. Inflammatory foci localized in the oral cavity of pregnant women may be a cause of preterm delivery, low birth weight (LBW), and preeclampsia, which may endanger both the mother’s and the child’s life [12,13]. Undoubtedly, hormonal changes that occur during pregnancy cause inflammation, but proper dental plaque control may minimize its risk, consequently limiting labor complications [14]. It has been confirmed that environmental factors affect the foetal development. They influence premature birth, impaired intrauterine growth, infant mortality or congenital birth defects [15]. The list of these factors includes smoking, excessive use of video display terminals, anesthetic gases, antineoplastic drugs and exposure to lead, selenium and inorganic mercury. Low birth weight and preterm delivery may be caused by: air pollution, pesticide exposure and stress [15,16]. It is crucial to identify environmental factors in the oral cavity that may have an impact on the pregnancy. This study aimed to assess the oral hygiene and periodontal status and their relationship with the occurrence of preterm delivery, low birth weight, and type of labor.

## 2. Materials and Methods

### 2.1. Materials

A total of 50 pregnant women were examined in Academic Centre of Dentistry of Silesian Medical University in Bytom, Non-Public Health Care ‘Lexton’ and Non-Public Health Care ‘Femina’ in Wodzislaw Slaski. Patients were randomly divided into two groups: B1 and B2; each group was composed of 25 patients (the research project obtained approval from the Bioethical Commission of the District Medical Board in Katowice; resolution no. KNW/0022/KBI/72/I/of 06.07.2010).

The B1 group was composed of 25 pregnant women who, in addition to a dental examination, were given instructions on how to properly maintain oral hygiene on a satisfactory level during that very special period of their life. The instructions included information on the proper method of brushing teeth (which was additionally presented on a phantom model). Additional products were recommended, such as dental floss, interdental toothbrushes, and an alcohol-free mouthwash that was chlorhexidine free and contained octenidine. All the instructions were given to the patients in a written form so they could review them whenever they wanted.

The B2 group comprised 25 patients who underwent dental examination but did not receive any instructions regarding oral hygiene maintenance.

Inclusion criteria:

Pregnant women (14–17 weeks of pregnancy) aged more than 18 years who provided written approval to participate in the study.

Exclusion criteria:

Pregnant women who did not provide written approval to take part in the research, were incapacitated, had underwent orthodontic treatment, were toothless, had not enough teeth to assess the indices used in the research, or who had temporomandibular junction dysfunction.

### 2.2. Methods

The patients were examined twice. The first examination (A_1_) was performed between 14 and 17 weeks of pregnancy, while the second examination (A_2_) was performed at the beginning of the third trimester, between 27 and 30 weeks of pregnancy.

The examination included an oral hygiene assessment and the following indexes were used [17]:

Proximal Plaque Index (API) by Lange: A yes/no decision was made based on whether the examined interproximal surfaces were covered by a plaque (+) or not (−). The index was calculated using the following formula:

(1) $$API=\frac{no.\;of\;plaque\;\left(+\right)\;sites}{no.\;of\;sites\;examined}\times 100\%$$

Depending on the results, the oral hygiene conditions of individuals were classified as follows:

100–70%—poor oral hygiene

69–40%—insufficient oral hygiene

39–25%—pretty good oral hygiene

<25%—optimum hygiene

Plaque Index (Pl.I.) by Silness and Loe: It measures the thickness of plaque on the gingival one-third of the teeth. The measurements were taken on six selected teeth: 16, 12, 24, 36, 32, and 44 on all (4) surfaces. The result of the examination was assigned a value between 0 and 3 [18]:

0 No plaque
1 A film of plaque adhering to the free gingival margin and adjacent area of tooth; the plaque may be seen in situ only after application of disclosing solution or by using a probe on the tooth surface
2 Moderate accumulation of soft deposits within the gingival pocket, or the tooth and gingival margin, which can be seen with the naked eye
3 Abundance of soft matter within the gingival pocket and/or on the gingival margin and adjacent tooth surface

First, the scores for individual teeth were calculated, then added, and finally divided by the number of assessed teeth. The possible results were rated from 0 to 3 (0, excellent hygiene; 0.1–0.9, good hygiene; 1.0–1.9, fair hygiene; and 2.0–3.0, poor hygiene).

Oral Hygiene Index-Simplified (OHI-S) by Green and Vermillion [17]: The index has two components: the Debris Index and the Calculus Index. Each of these indices is based on numerical determinations representing the amount of debris or calculus found on the preselected tooth surface. The six surfaces examined for the OHI-S were selected from four posterior and two anterior teeth. The examination was performed using a dental mirror. Six teeth were examined, including 16 and 26 on the buccal surfaces, 26 and 46 on the lingual surfaces, and 11 and 31 on the labial surfaces. The result of the examination was assigned a value between 0 and 3:

0 No debris or calculus
1 Soft debris or supragingival calculus, covering not more than one-third of the exposed surface
2 Soft debris or supragingival calculus, covering not more than two-thirds of the exposed tooth surface; presence of flecks of subgingival calculus around the cervical portion of the tooth; or both
3 Soft debris or supragingival calculus covering more than two-thirds of the exposed tooth surface, a continuous heavy band of subgingival calculus around the cervical portion of the tooth, or both

The results obtained for a particular tooth (surfaces) were added and then divided by the number of examined teeth. The possible results vary between 0 and 3 for the debris and calculus indices and between 0 and 6 for the OHI-S values.

To assess the periodontal tissues and gingiva, the following indices were used:

Muhlemann-Son Sulcus Bleeding Index (mSBI) was used to assess the severity of gingival bleeding. To estimate that index, a periodontal probe was passed along the gingival margin in order to provoke bleeding. The obtained results were scored with values from 0 to 3 [19]:

0 No bleeding on probing
1 Isolated bleeding spots
2 Blood formed a red line along the gingival margin
3 Heavy bleeding

Clinical Attachment Level (CAL): It is used to measure the position of the soft tissue in relation to the cementum-enamel junction (CEJ). To calculate CAL, two measurements were taken: the probing depth and the distance from the gingival margin to the CEJ. The probing depth plus the distance from the gingival margin to the CEJ comprised the clinical attachment level:

(2) $$\mathrm{CAL}=\frac{no.\;of\;sites\;with\;clinical\;attachment\;lost}{no.\;of\;examined\;sites}\times 100\%$$

The Community Periodontal Index of Treatment Needs (CPITN) makes it possible to record the most common periodontal conditions such as periodontal pockets, inflammation of the gingiva, or dental calculus. It enables the planning and monitoring of the effectiveness of periodontal treatment. Before the calculation, dentition was divided into sextants: 17–14, 13–23, 24–27, 34–37, 33–43, and 44–47. All teeth in each sextant were examined, and the highest value for the sextant was noted. After the clinical assessment, the following scores were given:

0 Healthy periodontal tissues
1 No pockets, calculus, or overhangs of fillings; bleeding occurred after probing in one or several units
2 No pockets > 3 mm, dental calculus, and plaque-retaining factors (overhanging fillings, crowns) visible
3 3.5–5.5 mm deep pockets; bleeding on probing
4 6 mm deep or deeper pockets

The treatment needs based on the score obtained are presented in Table 1.

After the diagnostic test, the B_1_ subgroup was given professional instruction on oral hygiene maintenance and received dental floss, interdental toothbrushes, and a mouthwash, which was alcohol and chlorhexidine free and contained octenidine. During the second appointment (after 3 months), a dental examination was performed to evaluate the same indices. After the theoretical date of labor, the patients were called in order to gather the following data: date and method of labor (natural vaginal delivery or Caesarian section). The patients were further asked about the sex and weight of their newborn.

### 2.3. Statistical Analysis

The quantitative variables (API, Pl.I, OHI-S, mSBI, CAL and CPITN, date of labor, weight of newborn) were analyzed using the Shapiro–Wilk test to verify whether these variables have a normal distribution. Student’s t-test was used to analyze dependent variables with a normal distribution, while Wilcoxon test was used to analyze variables with non-normal distribution. The qualitative variables (sex, type of delivery) were analyzed with the χ ^2^ test. The rho Spearman test was performed to verify the correlation between the qualitative variables. A *p* value of *p* < 0.05 was considered significant (Statistica 9.0, SUM, Katowice, Poland).

## 3. Results

The median age of B_1_ patients was 27 years, while that of B_2_ patients was 28 years. Most of the patients in each group had completed secondary education or had an academic degree. Most of the patients in both groups had no general disease (B_1_, 84%; B_2_, 80%). In both groups, three patients (12%) with hypothyroidism were treated with Euthyrox. One patient (4%) in the B_1_ group had asthma and was treated with Pulmicort. In the B_2_ group, one patient (4%) with systemic sclerosis was treated with metyperol, while another patient with ulcerative colitis was treated with Asamox and Pentasa. The statistical analysis showed a similar distribution of education and general disease occurrence between groups B_1_ and B_2_ (*p* < 0.05).

Results of the analysis of oral hygiene indices proved that during the first and second appointments, the API values were 40–60%, which indicated insufficient oral hygiene in both the B_1_ and B_2_ groups, thus requiring improvement in this area. The average values of Pl.I. in both groups during the first appointment were 1.1–2, which indicated poor oral hygiene. Statistically significant lower values of indices were observed in the B_1_ group during the second appointment (*p* < 0.05) (Table 2).

In order to assess the status of patient’s oral hygiene, the OHI-S Index by Green and Vermilion and its components were used: Dental Plaque Index [DI] + Calculus Index [CI] = OHI. The average values of the OHI-S index in both groups from the first appointment ranged from 1.3 to 3, which indicated that the patients’ oral hygiene was satisfactory. The results of the B_2_ group in the second appointment were similar to those in the first appointment. In the B_1_ group, the values of the OHI-S index in the second appointment were much lower (0.1–1) than those in the first appointment, which indicated that the group’s oral hygiene was good. The average values of DI_A1_ and CI_A2_ were also lower in the B_1_ group. The results were statistically significant (*p* < 0.05) for both DI and CI.

In the B_2_ group, which comprised patients who were not given any instructions about oral hygiene maintenance during the first appointment (A_1_), statistically higher values of DI_A2_ in comparison to DI _A1_ were observed; however, it did not cause any statistically significant changes in OHI_A2_ values (Table 3).

The results of the periodontal tissue assessment showed that the average value of mSBI in the B_1_ and B_2_ groups during both appointments (A_1_ and A_2_) ranged from 20% to 50%, which indicated moderate gingivitis. In both groups, the average values of mSBI at the second appointment were higher. In the B_2_ group, there was a statistically significant difference between mSBI_A1_ and mSBI_A2_ (*p* = 0.000018). In the B_1_ and B_2_ groups, the average CAL during the first appointment was 5%, while that during the second appointment was 6%. The probing depth was 5–6 mm, which indicated slight and moderate periodontitis. Results of the Wilcoxon test showed that there was no statistically significant difference between CAL values on the first and second appointments for both the B_1_ and B_2_ groups (*p* < 0.05) (Table 4).

During the first appointment (A_1_), most of the examined patients were given a CPI 2 score (i.e., presence of dental calculus) and CPI 3 score (pockets of depth 3.5–5.5 mm). Only one of the 25 patients in the B_2_ group was given a CPI 0 score. During the second appointment, the number of patients with CPI = 2 decreased (B_1T1_: 56% vs. B_1T2_: 26%) in favor of the CPI = 1 (B_1T1_: 20% vs. B_1T2_: 50%). None of the patients, during the second appointment, had healthy periodontal tissues. There was no change in the number of patients with CPI = 3 in either group (B_1T1T2_: 24% vs. B_2T1T2_: 28%).

The highest percentage of patients in both groups was classified as TN II according to treatment needs: oral hygiene instructions, scaling and root planning, and removal of plaque retentive factors. Approximately 80% of the patients from the B_1_ group and 64% from the B_2_ group were present during the first appointment; by contrast, only 50% of the patients from the B_1_ group and 68% from the B_2_ group were present during the second appointment.

All patients were contacted (after the planned date of labor predicted by the gynecologist based on the date of last menstruation and ultrasound results) in order to obtain information about the exact date of labor (pregnancy week), sex, and weight of the newborn. The patients were asked further regarding the method of labor (vaginal delivery or cesarean section).

**Figure 1:**
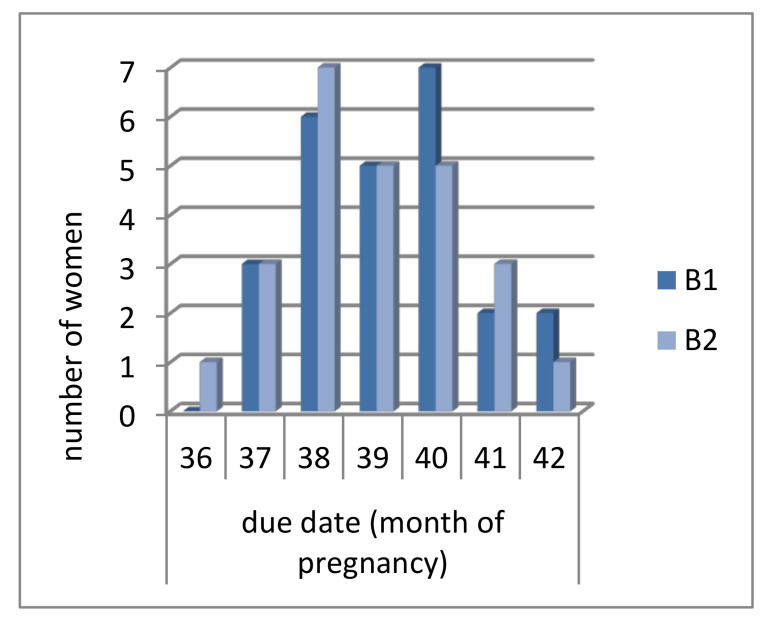
Due date of patients from B1 and B2 group.

In the B_1_ group, most of the patients delivered a baby in the 40th week of pregnancy (seven patients, 28% of the group); in the B_2_ group, most of the patients delivered a baby in the 38th week of pregnancy (seven patients, 28% of the group). Results of the Student’s *t*-test showed that there were no significant differences between the subgroups according to labor term (*p* = 0.853) (Figure 1).

The average newborn weights in both the B_1_ and B_2_ groups were similar (3200 g). There were no statistically significant differences in newborn weight between the two groups (*p* > 0.05). (Table 5)

**Figure 2:**
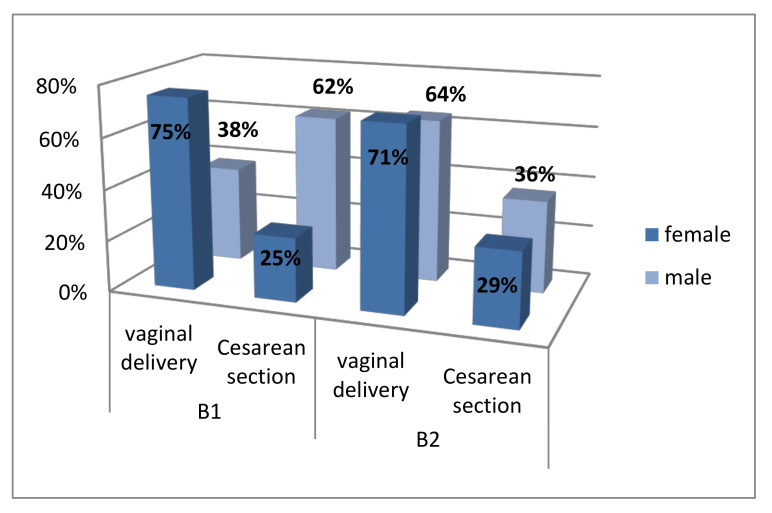
Distribution of vaginal deliveries and Cesarean sections between groups.

A total of 14 patients (56%) from B_1_ group and 17 (68%) from B_2_ group delivered their newborn via vaginal delivery. The rest of the patients, 11 in B_1_ and 8 in B_2_, underwent Cesarean sections. Of the patients from the B_1_ group who delivered via vaginal delivery, 75% had a female baby, while 38% had a male baby. Those in the B_2_ group, 71% had a female baby, while 64% had a male baby. The results of the χ^2^ test showed no statistically significant differences in the distribution of vaginal deliveries and Cesarean sections between groups (*p* = 0.382; Figure 2).

Assessment of the correlation between gingiva and periodontal tissue condition and date of birth, type of delivery, and weight of newborns showed a negative correlation between the mSBI and CAL indices and the period of child delivery. The results of both groups were statistically significant: B_1_: *p*_mSBI_ = 0.031, *p*_CAL_ = 0.009; B_2_: *p*_mSBI_ = 0.008, *p*_CAL_ = 0.001. The correlation coefficient r was −0.4 to −0.6, which showed that the correlation between the variables was moderate. The results indicated that in women who developed gingivitis and periodontitis (higher values of mSBI and CAL), the delivery occurred earlier than the planned date of childbirth. There was no statistically significant correlation between the mSBI and CAL indices and weight of the newborns (*p* > 0.05). There was a moderate positive correlation between Cesarean section in B_1_ patients and the intensity of inflammation. The correlation was statistically significant (*p* < 0.05) (Table 6).

## 4. Discussion

For many years, it has been discussed that it is crucial to educate patients on the importance of oral hygiene and its impact on general health [20]. It has been emphasized that cavities and any inflammatory foci located in the oral cavity are classified as foci that influence other organs and systems in the human body. Pregnancy is a very special period in women’s lives, in which many efforts are made to ensure that the future mother remains healthy and that the fetus develops properly. A pregnant woman should be carefully taken care of by a group of specialists, including a dentist. Several published studies showed that any inflammation found in the oral cavity as well as the diversity of bacterial flora, such as anaerobic bacteria Gram-negative bacteria, may be increase the possibility of premature births, low birth weights, preeclampsia, and intrauterine infections [21,22]. Prophylactic interventions performed during pregnancy may decrease the number of cariogenic bacteria as well as limit their transmission and growth in the newborn oral cavity [22].

Our study proves that the status of oral hygiene in Polish pregnant women is unsatisfactory. The analysis of oral hygiene with the use of Pl.I. confirmed that the aforementioned women had poor oral hygiene during the first dental appointment. Similar observations have been described by Moreir et al. who observed improvement in oral hygiene status after providing prophylactic and therapeutic treatments [23]. To assess the oral hygiene status, the API index was used as well. The index was rated based on the absence or presence of dental plaque on the proximal surfaces of the teeth, and the results obtained were 40% to 60%, which indicated insufficient oral hygiene. There was a decrease in the index values after the professional instructions regarding oral hygiene were given and the dental floss and Octenidol mouthwash were recommended. Nakonieczna-Rudnicka et al. assessed the oral hygiene status of pregnant women who smoke. She proved that most of the smoking pregnant women had insufficient (50%) and bad (31.03%) oral hygiene, while non-smoking pregnant women showed optimal oral hygiene (24.14%) and insufficient (22.58%) [24]. The results of the analysis of the OHI-S index by Green and Vermilion and its components in both groups demonstrated satisfactory oral hygiene. Higher values of OHI-S during the first appointment were strictly connected (in authors opinion) to the calculus that was extensive. A statistically significant difference was observed in the CI values in the B1 group between the first and second appointment [25]. In a previous Indian study conducted in 300 pregnant women, Gupta et al. showed that 40.7% of women had satisfactory oral hygiene, while 44% had bad oral hygiene; these were findings observed in women in the second and third month of pregnancy. Furthermore, the study demonstrated a medium OHI-s value of (1.68, 4.26) 2.97 ± 1.29 (DI: (1.01, 2.17)1.59 ± 0,58; confidence interval: (0.51, 2.05) 1.28 ± 0.77) [26]. Undoubtedly, the lower index values indicating the status of oral hygiene observed during the second appointment were strictly correlated to the hygienic instructions provide to the patients [27].

In the study, the influence of steroid hormones (estrogen and progesterone) is widely discussed and confirmed. In 2010, in Spain, the influence of higher concentrations of steroid hormones on the gingiva and periodontal tissues was reported. Figuero et al. observed an increase in Gingival Index (GI) in the third trimester of pregnancy in women who, before the examination, were classified as having a proper clinical picture of periodontium and proper oral hygiene [28]. Similar results were obtained by Borgo et al., who conducted a study in Brazil. They described that the Gingival Bleeding Index in pregnant women in the third trimester of pregnancy was twice as high as that in pregnant women in the second trimester, and was five times lower than the values in patients who were not pregnant but had similar clinical picture of oral hygiene [29]. These results may confirm the assumption made by the American Academy of Periodontology that the presence of gingivitis gravidarum is associated with the general factor of pregnancy and the fluctuations in steroid hormone levels. In our study, redness, edema, and bleeding in the gingiva were observed in patients at the beginning of the second trimester of pregnancy, *while moderate gingivitis* was observed in both groups, which confirms the similarities in mSBI values between groups (mSBI_B1_ = (10.59, 65.17) 37.88 ± 27.29; mSBI_B2_ = (7.14, 71.18)39.16 ± 32.02) during the first appointment. Based on the definition of gingivitis gravidarum, the association between the presence of dental plaque and inflammation in the oral cavity should be emphasized. The results of Moreir et al. demonstrated the risk of gingivitis in pregnant women with dental plaque. They observed a decrease in gingival index medium values after an 8-week-long break between examinations, during which the amount of dental plaque was controlled by non-surgical treatments (scaling above and under the gingiva) [30]. These observations confirm that gingivitis is correlated with the level of steroid hormones, the increase of which causes edema of the tissues due to the increased permeability of cell membranes. They also confirmed that proper performance of hygienic procedures may help maintain a healthy gingiva despite the changes occurring during pregnancy and the increase in hormone concentration [31]. Moreover, although pregnant women have higher risk of developing gingivitis, clinical attachment loss (CAL) was not observed. In our study, an increase in CAL values was not observed in pregnant women (probing depth: 5–6 mm). Gursoy et al. observed a statistically significant difference in the number of patients with dental pockets of 4 mm and deeper in 25–27 weeks of pregnancy and 34–38 weeks of pregnancy in comparison to those in the first trimester of pregnancy, in childbirth, or lactating mothers [32]. Borgo et al. had similar observations; additionally, they described a higher number of *Actinobacillus actinomycetemcomitans* in pregnant women, suggesting that changes in bacterial flora may cause more advanced inflammatory processes in pregnant women [29].

Dental plaque and any pathologies commonly occur in the periodontal tissues, which are the habitat of anaerobic Gram-negative bacteria whose presence triggers immunologic responses with production of cytokines IL-1, IL-6, and TNF-α, and prostaglandins [23,33]. It was in the 1940s when Galloway proved the influence of anaerobic bacteria on preterm labor (PTL, which takes place before the 37th week of pregnancy) and LBW (<2500 g). The present study has not confirmed this correlation; however, we observed a correlation between the week of labor and the values of mSBI. Seven pregnant women (14%) delivered a baby at 36th–37th week of pregnancy, 86% of whom were diagnosed with moderate and severe and generalized gingivitis. However, none of the newborns had LBW. Similar observations were reported by Karimi et al. [34]. They suggested that Cesarean section may be a risk factor for low birth of newborns. This kind of correlation was not observed in our study; approximately 25% of B1 and 29% of B2 patients underwent Cesarean section. Our results were higher than those reported in a study conducted in Turkey in 2009 by Toygar et al., but lower than those observed by Janus in 2010, who noted 33.9% of Cesarean sections [1,35]. These results may be associated with the fact that nowadays more and more women prefer to undergo Cesarean section. Toygar observed a higher risk of LBW in newborns delivered through Cesarean section, emphasizing their iatrogenic character. He noticed that the assessment of the gingiva and periodontal tissues of pregnant women performed by a midwife during the first appointment may be an indicator of the risk of complications such as PLT and LBW in patients with higher CPITN values. He did not assess the correlation between the type of labor and periodontium condition, which was assessed in one study conducted in B1 patients who had Cesarean section with higher mSBI values. Toygar also pointed out that periodontal diseases are a greater risk factor for LBW [35]. *Gesea* et al., in their research conducted in Tanzania, observed that periodontal diseases are potential independent risk factors for LBW, PTL, and pre-eclampsia [36].

Regarding limitations of the work, one of the common difficulties in scientific research is the determination of suitable sample size. Calculations of the sample size are necessary to validate the test designs and justify the authenticity of the test results. The determination of the sample size for testing is a key element. There are many situations where sample size is determined depending on the specificity of the study and potential difficulties in recruiting participants [37]. Pregnant women are a special, sensitive group of study participants. In the presented study, the difficulties in gathering a large number of respondents confirm this problem. Despite the need for relatively regular gynaecological visits, pregnant women did not always show the need to intensify their oral examination. This is confirmed by the reports of other authors, who, like our research team, point out that pregnant women often do not appear at the next visits planned in the research project, when visit are not directly connected with the analysis of pregnancy status [38]. In such situations, the initially higher number of respondents falls significantly lower than initially expected. When results are statistically significant and important for subsequent studies in small groups, and pregnant women are always included in this important group, authors should strive to publish the results and take part in discussions with other researchers.

## 5. Conclusions

Results of the analysis of the given data indicate that gingivitis commonly occurs in pregnant women. The more advanced the pregnancy, the more acute the inflammation, which is the risk factor influencing the date of labor. Doubtfully, the novelty of the study is investigating the association between dental hygiene during pregnancy and preterm birth. The present study did not show such an association, however, we observed a correlation between the gestational weeks at labor and the values of mSBI, which in dental examinations is one of the indicators used in oral health analysis also in relation to hygiene.

The pregnant women showed improvement in oral hygiene after receiving professional instructions and care from the dentists. This finding indicates that specialists (including dentists) should provide interdisciplinary care to pregnant women and information regarding the influence of oral diseases on them and their newborn.

The authors confirmed that the results correspond well with scientific literature on the condition of the oral cavity in pregnant women and believe that further studies on larger samples in this area are necessary to draw further conclusions that will contribute to improving the condition of the oral cavity of pregnant women, especially in post-industrial areas where such a significant number of external factors affect the course of pregnancy and the condition of newborns.

## Figures and Tables

**Table 1:** Category of treatment needs.

Clinical Score	Category of Treatment Needs
0 1	I. Improvement of personal hygiene (oral hygiene instructions)
2 3	II. Oral hygiene instructions. Scaling and root planning. Removing of plaque retentive factors
4	III. Oral hygiene instructions. Scaling and root planning. Removal of plaque retentive factors. Complex treatment

**Table 2:** Results of the Student’s *t*-test or Wilcoxon test for API and Pl.I.

Group	Appointment	Parameters				Student *t*-Test	Wilcoxon Test
		Mean	SD	Δ Mean	Δ SD	*p*	*p*
		API					
B_1_	A1	55.92	26.55	10.28	15.37	0.003	-
	A2	45.64	21.07				
B_2_	A1	44.48	29.05	−0.94	6.71	0.491	-
	A2	45.42	28.35				
		Pl.I.					
B_1_	A1	1.24	0.89	0.44	0.66	-	0.001
	A2	0.81	0.76				
B_2_	A1	1.19	0.89	−0.03	0.18	-	0.391
	A2	1.22	0.96				

**Table 3:** Results of the Wilcoxon test for the OHI-s index and its components: DI and CI.

Variables		Appointment	Parameters				*Wilcoxon* Test
			Mean	SD	Δ Mean	Δ SD	*p*
			B_1_				
OHI-s	DI	A1	1.13	0.8	0.27	0.4	0.004
		A2	0.86	0.66			
	CI	A1	0.57	0.62	0.45	0.59	<0.001
		A2	0.12	0.26			
	DI + CI	A1	1.7	1.29	0.72	0.72	<0.001
		A2	0.98	0.86			
			B_2_				
OHI-s	DI	A1	1.11	0.88	−0.08	0.19	0.027
		A2	1.19	0.84			
	CI	A1	0.58	0.62	−0.01	0.34	0.97
		A2	0.59	0.59			
	DI + CI	A1	1.7	1.38	−0.09	0.37	0.085
		A2	1.79	1.27			

**Table 4:** Results of Student’s *t*-test and Wilcoxon test for mSBI and CAL indices.

Group	Appointment	Parameters				Student’s *t*-Test	Wilcoxon Test
		Mean	SD	Δ Mean	Δ SD	*p*	*p*
		mSBI					
B_1_	A1	47.66	27.29	1.10	9.55	0.570	-
	A2	46.56	26.35				
B_2_	A1	39.16	32.02	−10.52	8.37	-	<0.001
	A2	49.68	30.9				
		CAL					
B_1_	A1	4.52	9.18	−0.44	0.94	-	
	A2	4.96	8.24				
B_2_	A1	5.20	9.56	−0.48	0.96	-	
	A2	5.68	8,60				

**Table 5:** Parameters of descriptive statistics and results of Student’s *t*-test for newborn weight in groups B_1_ and B_2._

Variable	Group	Parameters of Descriptive Statistics				Student’s *t*-Test
		Mean	SD	Minimum	Maximum	*p*
Newborn weight (g)	B_1_	3292.40	246.92	2810.00	3900.00	0.679
	B_2_	3261.20	281.15	2670.00	3780.00	

**Table 6:** Results of the correlation analysis between mSBI and CAL indices and date of birth, newborn weight, and type of delivery in B_1_ and B_2._

Correlation	B_1_		B_2_	
	r	*p*	r	*p*
mSBI—date of birth	−0.43	0.031	−0.52	0.008
mSBI—newborn weight	−0.35	0.085	−0.13	0.522
mSBI—type of delivery	0.45	0.023	−0.02	0.933
CAL—date of birth	−0.51	0.009	−0.63	0.001
CAL—newborn weight	−0.39	0.055	−0.23	0.259
CAL—type of delivery	0.27	0.189	−0.32	0.116
